# Paeonol-Loaded Ethosomes as Transdermal Delivery Carriers: Design, Preparation and Evaluation

**DOI:** 10.3390/molecules23071756

**Published:** 2018-07-17

**Authors:** Hongdan Ma, Dongyan Guo, Yu Fan, Jing Wang, Jiangxue Cheng, Xiaofei Zhang

**Affiliations:** 1 Shaanxi Province Key Laboratory of New Drugs and Chinese Medicine Foundation Research, Shaanxi University of Chinese Medicine, Xianyang 712000, Shaanxi, China; mahongdancy@163.com (H.M.); fans95950yu@163.com (Y.F.); wangjing19890126@126.com (J.W.); cjx511@sntcm.edu.cn (J.C.); zhangxiaofei830@163.com (X.Z.); 2 Department of Pharmaceutics, College of Pharmacy, Shaanxi University of Chinese Medcine, Xianyang 712000, Shaanxi, China

**Keywords:** paeonol, ethosome, transdermal delivery, encapsulation efficiency, zeta potential, optimized formulation

## Abstract

Paeonol exhibits a wide range of pharmacological activities, such as anti-inflammatory, antidiabetic as well as pain-relieving activities. However, its intrinsic properties, such as low water solubility, poor stability and low oral bioavailability, restrict its clinical application. The current study aimed to optimize paeonol-loaded ethosomal formulation and characterize it in terms of encapsulation efficiency (EE), vesicle size (VS), zeta potential (ZP) and polydispersity index (PDI), in addition to differential scanning calorimetry (DSC), X-ray diffraction (XRD) and Fourier-transform infrared spectroscopy (FT-IR) studies. Here, paeonol-loaded ethosomes were prepared by the injection method and optimized by the single-factor test and central composite design-response surface methodology. The optimized paeonol-loaded ethosomes had an EE of 84.33 ± 1.34%, VS of 120.2 ± 1.3 nm, negative charge of −16.8 ± 0.36 mV, and PDI of 0.131 ± 0.006. Ethosomes showed a spherical morphology under the transmission electron microscope (TEM). DSC, XRD and FT-IR results indicated that paeonol was successfully incorporated into the ethosomes. In-vitro transdermal absorption and skin retention of paeonol from paeonol-loaded ethosomes were 138.58 ± 9.60 µg/cm^2^ and 52.60 ± 7.90 µg/cm^2^, respectively. With reasonable skin tolerance, ethosomes could be a promising vehicle for transdermal delivery of paeonol.

## 1. Introduction

Paeonol (2′-hydroxy-4′-methoxyacetophenone) is the main active component found in peonies such as Paeonia moutan and Radix Cynanchi Paniculati [1]. Studies on the pharmacological activities of paeonol have proven their anti-inflammatory [2], antidiabetic [3], antiallergic [4] and antitumor [5] activities, besides their ability to improve cognitive impairment [6]. Therefore, paeonol is a valuable drug with promising prospects. However, oral administration of paeonol is limited by its low bioavailability, low water solubility [7] and poor stability [8]. In order to address these limitations, transdermal drug delivery systems (TDDSs) of paeonol were designed and prepared. TDDSs provide significant advantages, since they avoid first-pass metabolism, maintain plasma concentration, and reduce dosing frequency [9]. With the development of medical technology, ethosomes have been shown to play an important role in TDDSs. Unlike conventional liposomes, ethosomes could effectively transport drugs through the stratum corneum (SC) into deeper layers of skin, even into the circulating blood [10]. Therefore, they have attracted wide attention in recent years [11]. Ethosomal formulations were prepared to improve the bioavailability of raloxifene hydrochloride [12]; and griseofulvin (GRF)-loaded ethosomes were designed for dermatophytoses, results of which proved the GRF formulation to be safe and effective [13]. In our study, paeonol-loaded ethosomes (abbreviated as PAE ethosomes henceforth) were prepared for transdermal delivery of paeonol. The single-factor test and central composite design-response surface methodology were employed to optimize the ethosomal formulations. Physicochemical characteristics of the PAE ethosomes, including encapsulation efficiency (EE), vesicle size (VS), zeta potential (ZP) and polydispersity index (PDI) were determined, besides conducting differential scanning calorimetry (DSC), X-ray diffraction (XRD) and Fourier-transform infrared spectroscopy (FT-IR) studies. Moreover, in-vitro skin permeation and deposition of PAE ethosomes were assessed, along with skin irritation studies.

## 2. Results

### 2.1. Optimization of Paeonol Ethosomal Formulation

As shown in Table 1, the single-factor test was used to study the effect of several factors on EE, ZP, VS and PDI, including the concentrations of ethanol, amount of cholesterol (CHO) and soybean phosphatidylcholine (SPC), and so on. Table 2 shows independent variables and the corresponding levels in central composite design. A 17-run experiment was designed to study the influence of three factors and three levels on the response variables. The results are presented in Table 3. Regression analysis was performed for determining the optimal region for response studies. The final empirical models in terms of coded factors are shown in Equation (1),

Y = 0.72 + 0.15A + 0.095B + 0.28C + 0.062AB − 0.068AC + 0.077BC − 0.013A^2^ − 0.22B^2^ − 0.22 C^2^, (*p* < 0.05). (1)

**Figure 1:**
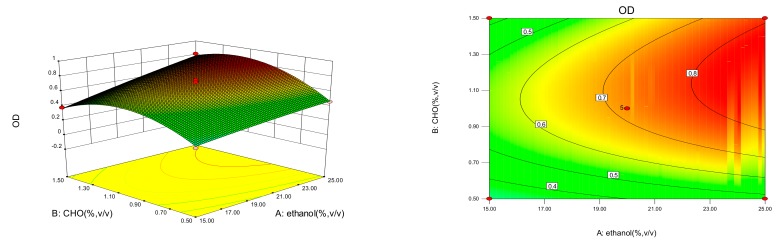

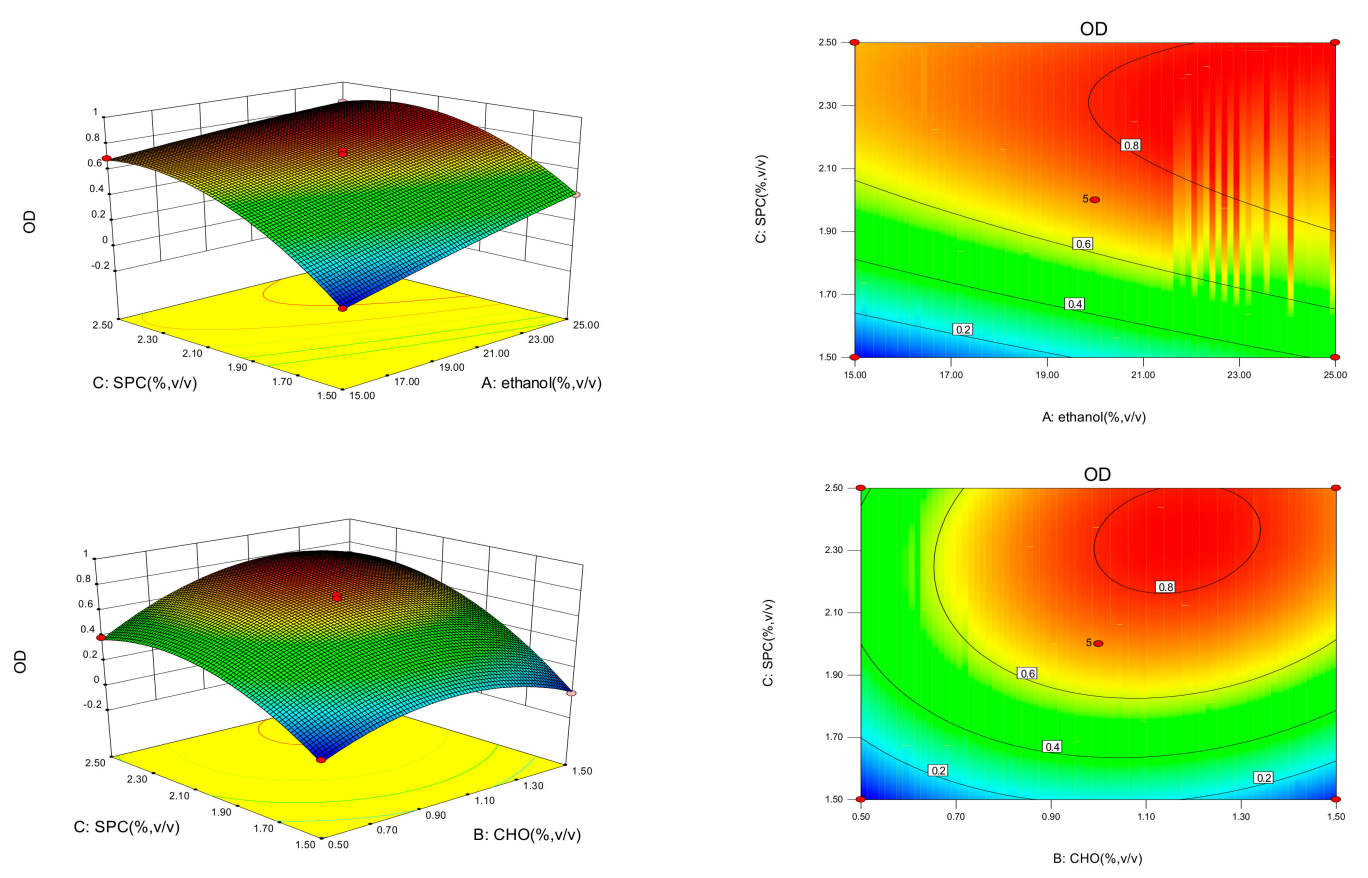
The response surface and contour plots of the fitting equations of the concentrations of ethanol (A), CHO (B) and SPC (C) on OD (Y).

A comprehensive evaluation of all the factors and levels is shown in Figure 1. Statistical analysis of the experimental results was evaluated by the software Design-Expert 8.0.6. To achieve the maximum OD of 0.85, 25% (*v*/*v*) ethanol, 1.5% (*w*/*v*) CHO and 2.5% (*w*/*v*) SPC were determined as the optimal levels at which OD was found to be 0.84. The errors between the experimental and predicted results were found to be very small.

### 2.2. Characterization of PAE Ethosomes

**Figure 2:**
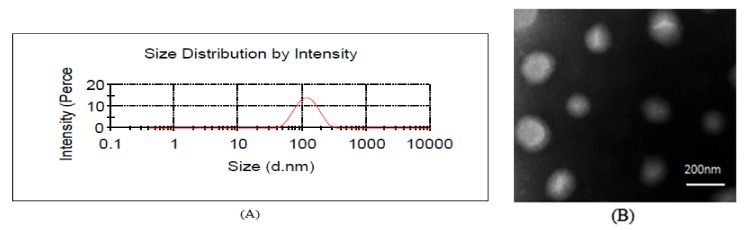
Particle size distribution (**A**) and surface morphologies (**B**) of PAE transethosomes by TEM.

The EE, ZP, VS and PDI of the PAE ethosomes are shown in Table 4. The EE of the PAE ethosomes was 84.33 ± 1.34%. The negative charge of ethosomes might be due to the effect of phospholipids and ethanol. The ZP of PAE ethosomes was −16.8 ± 0.36 mV, which could predict good stability of the formulation. The average size of PAE ethosomes was 120.2 ± 1.3 nm, which was appropriate to penetrate deep into the skin. The representative particle size diagram is shown in Figure 2A, with a narrow unimodal peak, indicating the relative homogeneity in size distribution of ethosomes. Figure 2B shows the ethosomes as nearly spherical vesicles with an average particle size of 105.5 ± 10.7 nm. The particle sizes of samples from the TEM images are smaller than the diameter measured by dynamic light scattering (DLS). These variations in the particle sizes are the result of the different measurement principles used by these two methods. This is in line with the literature [14].

**Figure 3:**
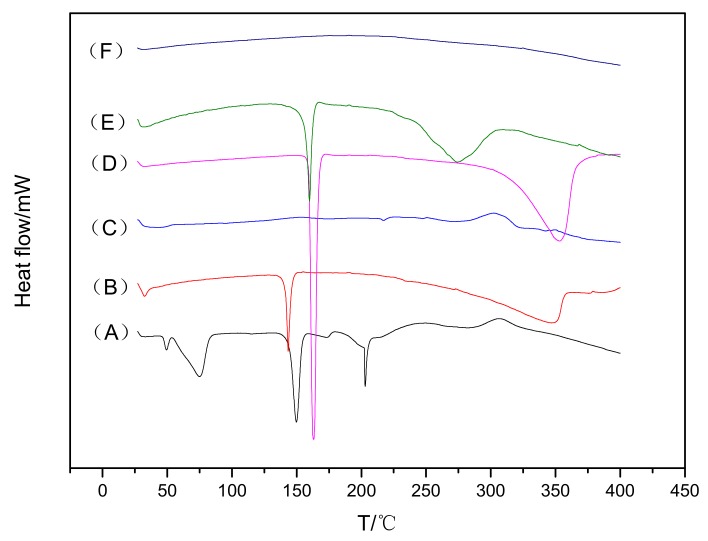
DSC analysis thermograms: (A) paeonol; (B) CHO; (C) SPC; (D) mannitol; (E) the physical mixture; (F) PAE ethosome freeze-dried powder.

DSC thermograms of pure paeonol, CHO, SPC, mannitol, their physical mixture, and freeze-dried powder of PAE ethosomes are shown in Figure 3. The DSC thermogram of pure paeonol exhibited a sharp endothermic peak at around 150 °C. A similar peak of pure paeonol was also seen in the thermogram of the physical mixture, which indicated feeble or no interaction at all. However, the peak disappeared in the case of ethosomes, implying the amorphous state of paeonol in ethosomes.

**Figure 4:**
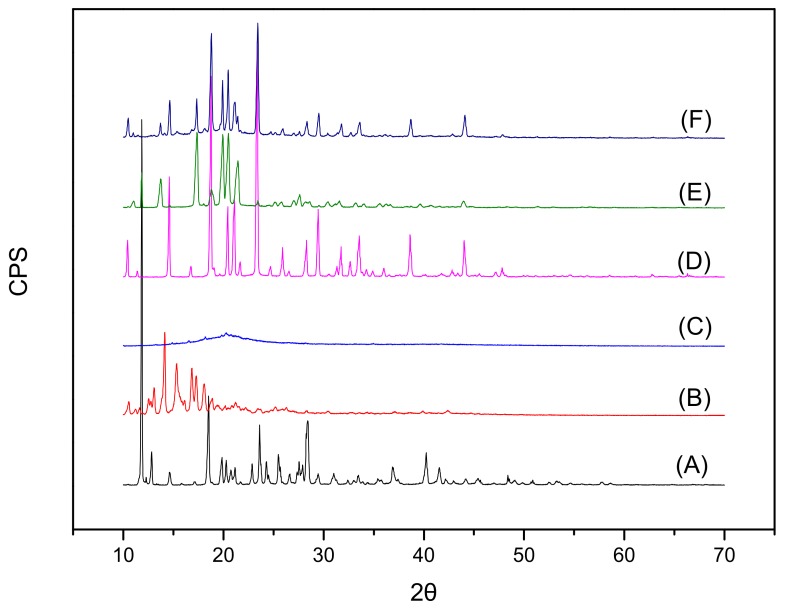
X-ray diffraction spectra: (A) paeonol; (B) CHO; (C) SPC; (D) mannitol; (E) the physical mixture; (F) PAE ethosome freeze-dried powder.

The XRD patterns of pure paeonol, CHO, SPC, mannitol, physical mixture, and freeze-dried powder are shown in Figure 4. Paeonol showed a strong diffraction peak at 11.83° (A), which also appeared in the spectra of the physical mixture (E), thereby suggesting that the crystal form of paeonol was the same as that in pure paeonol powder. In contrast, absence of the diffraction peak in the spectrum of freeze-dried ethosome powder (F) indicated its molecular or amorphous state, consistent with the results of DSC evaluation.

**Figure 5:**
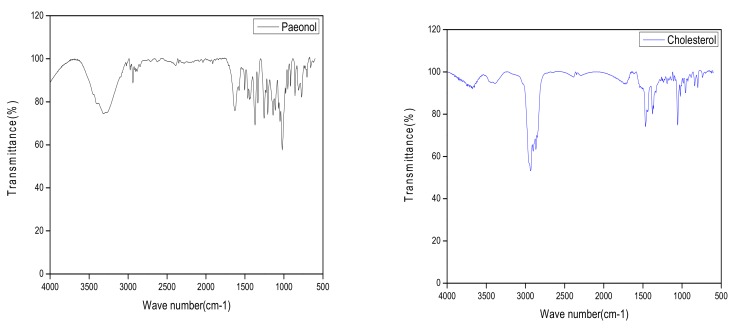

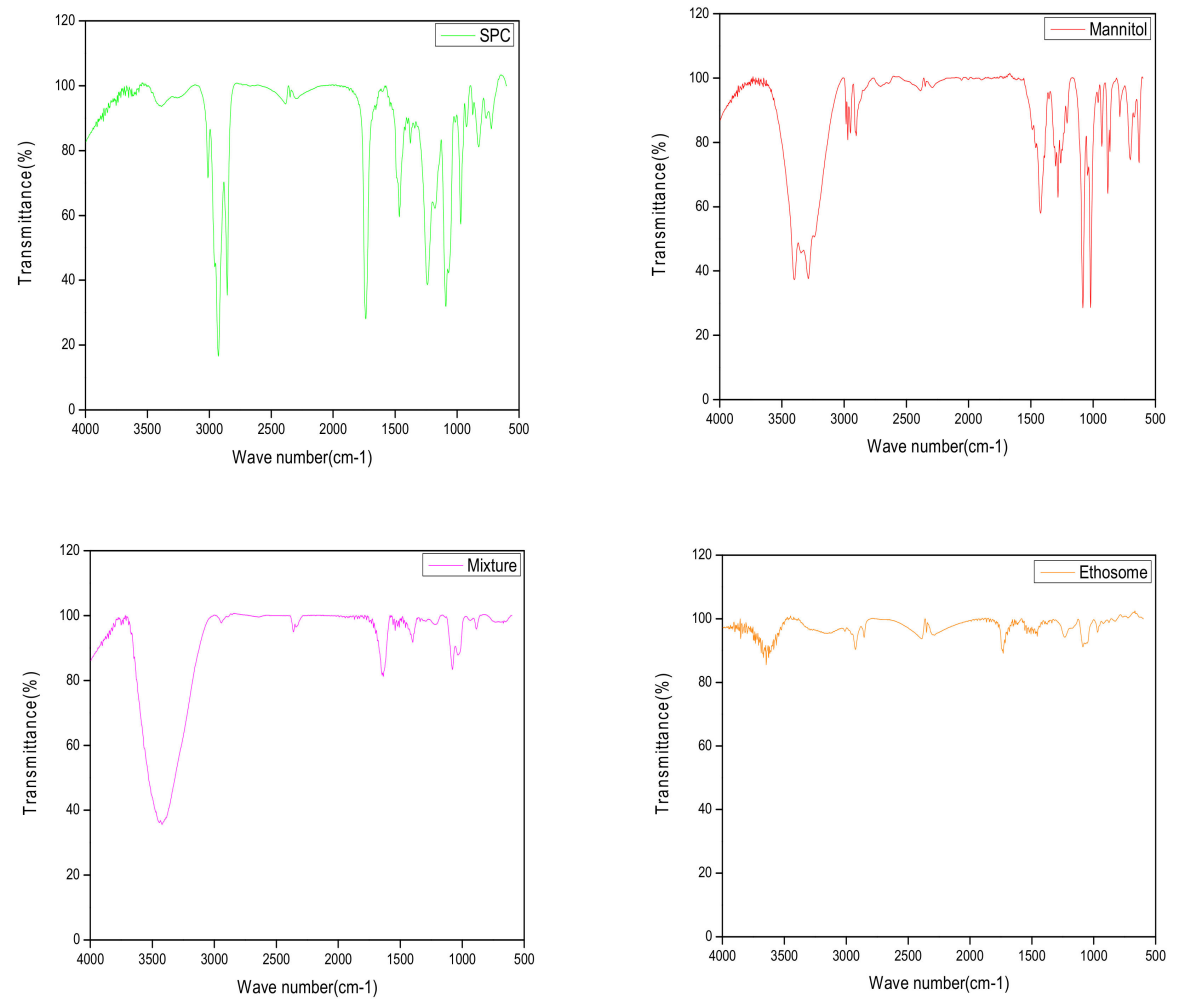
FT-IR spectra of paeonol, CHO, SPC, mannitol, the physical mixture and ethosome freeze-dried powder.

FT-IR spectra of pure paeonol, CHO, SPC, mannitol, physical mixture, and freeze-dried PAE ethosome powder are shown in Figure 5. While paeonol shows the characteristic FT-IR peak at 3342 cm^−1^, corresponding to O–H stretching, and the same was present in the physical mixture as well, it disappeared in ethosomes. These results demonstrated that paeonol had been successfully incorporated into the lipid matrix of ethosomes in amorphous or disordered states.

### 2.3. Validation of HPLC Method

The calibration curve for paeonol was linear in the range of 1.0–200.0 μg/mL; the regression equation being: y = 66.283x + 29.748 (r = 1). The relative standard deviation (RSD) values of intra- and interday precision were all below 5%. The intraday and interday accuracy varied between 98.9% and 103.1%. Recovery of paeonol, determined in high, medium and low concentrations, was 96.3%, 97.5%, and 102.6%, respectively, which indicated the values to be reliable and reproducible.

### 2.4. In-Vitro Transdermal Absorption and Skin Retention Studies

**Figure 6:**
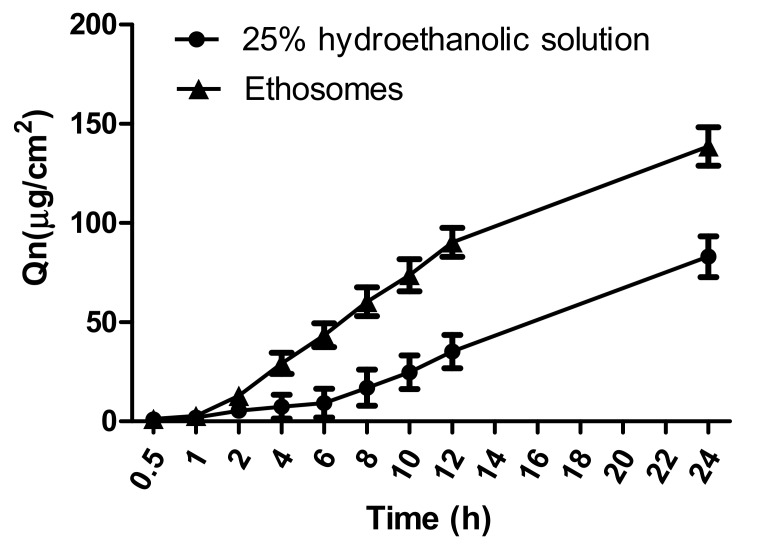
In-vitro permeation profiles of paeonol formulations through the rat skin (*n* = 3).

In-vitro permeation profiles of paeonol formulations through rat skin are shown in Figure 6. The cumulative penetration amount (Qn) of paeonol from 25% hydroethanolic solution was taken as control. A steady increase of paeonol in each formulation was observed during the 24 h period. Cumulative penetration amount (Qn) of paeonol from ethosomes (138.58 ± 9.60 µg/cm^2^) was significantly higher than that from 25% hydroethanolic solution (83.02 ± 10.30 µg/cm^2^) at 24 h.

**Figure 7:**
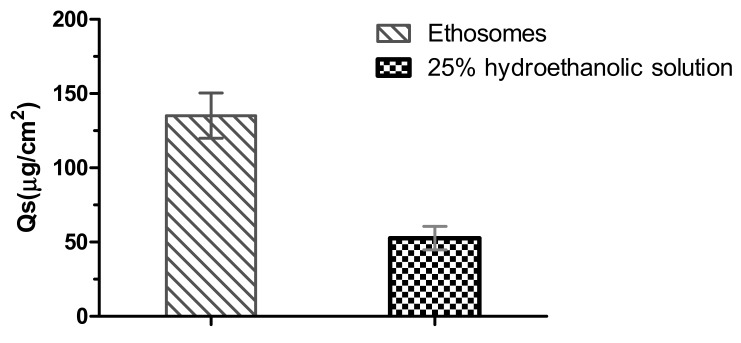
The deposition of the paeonol in the rat skin after in-vitro skin permeation experiment (*n* = 3).

Skin deposition (Qs) of paeonol was also calculated at the end of the permeation experiment (Figure 7). The Qs value of paeonol in the skin was significantly higher (135.14 ± 15.2 µg/cm^2^) for ethosomes, compared to that for 25% hydroethanolic solution (52.60 ± 7.90 µg/cm^2^) (*p* < 0.01).

### 2.5. Skin Irritation Study

**Figure 8:**
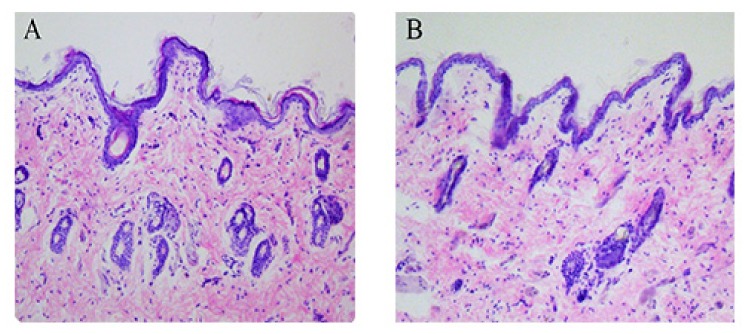
Skin cross-sections after staining with hematoxylin-eosin and examination under light microscope. (**A**): Control with no treatment, (**B**): PAE ethosomes.

Histopathological images of hematoxylin–eosin-stained cross-sections of skin are shown in Figure 8. Upon visual examination of rat skin for irritation due to PAE ethosomes, no sign of inflammation was observed. The cross-sections of ethanol-treated skin were not significantly different from those of untreated control skin samples. Therefore, the prepared PAE ethosomes were safe for skin application without any irritation.

### 2.6. Physical Stability Assay

**Figure 9:**
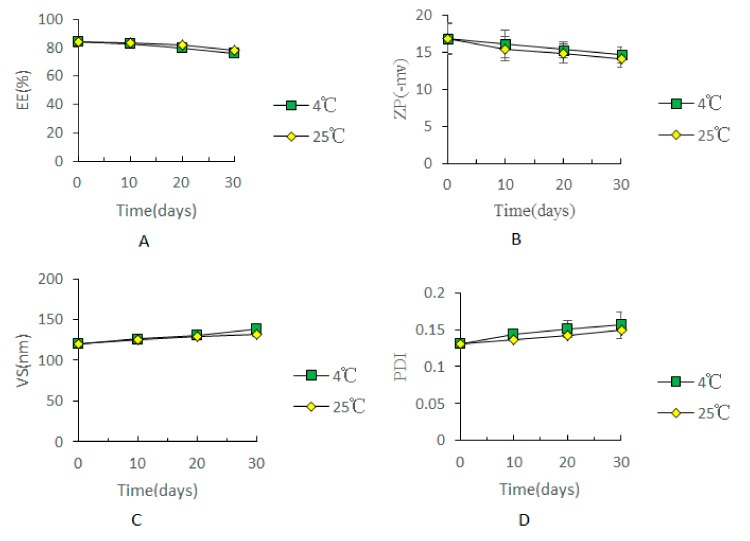
The effect on EE (**A**), ZP (**B**), VS (**C**) and PDI (**D**) stored at different temperatures: 4 ± 1 °C and 25 ± 1 °C for 30 days (*n* = 3).

The results of physical stability are presented in Figure 9; the EE, ZP, VS and PDI values of the optimized ethosome formulation at 4 ± 1 °C and 25 ± 1 °C are shown. During 30 days of storage, physical appearance and the above-mentioned parameters of the formulation remained almost unaltered at 4 °C and 25 °C. Compared to that at day 0, there was no significant difference in the values at days 10, 20 and 30 (*p* > 0.05), indicating an appreciable stability of the optimized ethosome formulation at 4 °C and 30 °C over 30 days.

## 3. Discussion

As a skin drug delivery system, ethosomes provide a new strategy with several advantages [15]. Ethosomes are commonly prepared by the ethanol injection method [16] and film hydration method [17]. Different preparative methods are selected according to the physicochemical properties of the drugs and experimental conditions, and each method imparts slightly different properties to the prepared ethosomes. Although the injection method is simpler, it needs to be improved owing to the numerous influencing factors. In this study, injection and ultrasonic methods were used to prepare PAE ethosomes. During the preparation, attention should be paid to the slow injection of water under sealed conditions, and the resulting ethosomes should be kept in a sealed manner to avoid ethanol volatilization. Although EE may be measured by a dialysis method [18], microcolumn centrifugation technique [18], ultracentrifugation method [19] or ultrafiltration centrifugation method [20], the first two techniques are very lengthy, and ultracentrifugation needs a 40,000-rpm high-speed freezer centrifuge. The smaller the particle size, the greater the speed required, and the longer the time and higher the equipment specifications; hence, the first three techniques were not selected for the study. In the present study, EE was determined by the ultrafiltration centrifugation method. Central composite design-response surface methodology was combined with the single-factor test to optimize the preparation procedure. Results showed that the optimized formulation had good stability, high entrapment efficiency, and small particle size. Here, the size measured by TEM was smaller than that measured by DLS. This was mainly due to different measurement principles used by these two methods. In the case of the TEM methods, the samples must be dehydrated and immobilized on a solid support. This can lead to structural distortions compared to the solvent-swollen state [14], whereas the size measured by DLS was a hydrodynamic diameter (hydrated state), and therefore the nanoparticles showed a larger hydrodynamic volume due to solvent effect in the hydrated state. DLS and TEM provide different types of information about particle size, and in some sense, they are complementary. The OD value was approximately equal to the predicted value, hence suggesting that the experimental design was reasonable and reliable.

Results of DSC, XRD and FT-IR experiments showed that the peaks in optimized formulations were diminished or suppressed, indicating successful incorporation of paeonol into the lipid matrix of ethosomes in amorphous or disordered states. Many researchers have reported similar results in which the peak intensity decreases and the amorphous state was confirmed [12].

In the in-vitro permeation study, cumulative permeation and skin deposition of paeonol, delivered from PAE ethosomes, through the skin was 1.6-fold and 2.5-fold higher, respectively, than that from 25% hydroethanolic solution (Figure 6). The results may be due to the structural similarity of ethosomes to the components of skin. A drug reservoir was created on the skin, retarding drug release, and enhancing skin retention. The skin irritation test is one of the important indicators for evaluating the safety of percutaneous preparations [21]. Results of skin irritation studies [22] verified PAE ethosomes as a safe carrier for transdermal delivery with no associated irritation. Physical stability after 30 days of storage at 4 ± 1 °C and 25 ± 1 °C, as determined from the EE, ZP, VS and PDI values, did not change significantly. The underlying reason for this observation may be that addition of ethanol causes a negative charge on the surface of PAE ethosomes, thereby making it difficult to cluster between particles [23,24]. Moreover, addition of cholesterol in the formulation can effectively increase the toughness of the phospholipid bilayer of PAE ethosomes [25], thus making the ethosomes have a smaller particle size and better stability; this also solved the problem of liposomal instability at room temperature.

## 4. Materials and Methods

### 4.1. Materials

Soybean phosphatidylcholine (SPC) was purchased from Macklin Biochemical Co., Ltd. and contained 98% phosphatidylcholine (Shanghai, China). Paeonol was procured from Xi’an Desheng yuan Biotechnology Co., Ltd. (purity: 99%, Xi’an, China). Cholesterol (CHO) was obtained from Macklin Biochemical Co., Ltd. (Shanghai, China). Mannitol was purchased from Tianjin Guangfu Fine Chemical Research Institute (Tianjin, China). The methanol and acetonitrile were of HPLC grade. All other chemicals used in the study were of analytical grade without further purification. Ultrapure water was used throughout the study. Male Sprague-Dawley rats (250 ± 20 g) were used and supplied by Chengdu Dashuo Animal Experiment Center. Rats were raised under standard temperature, humidity, and light conditions, as well as standard rodent diet and water. The research was approved by the Ethical Committee of Shaanxi University of Chinese Medicine.

### 4.2. Preparation of Paeonol Ethosomes

#### 4.2.1. Single-Factor Test

Paeonol ethosomes were prepared following an ethanol injection method with minor modifications [26]. In brief, required amounts of paeonol, SPC, and CHO were dissolved in approximately 3 mL of ethanol, and the aqueous phase slowly injected into ethanol by means of a syringe pump with constant stirring at 700 rpm at 25 °C for an hour, until the total volume increased to 10 mL. The single-factor test was carried out to optimize the preparation process.

#### 4.2.2. Optimization Using Central Composite Design-Response Surface Methodology (CCD-RSM)

CCD-RSM was chosen to optimize the preparation technique. The results of single-factor test are listed in Table 1, and details of the design are listed in Table 2. Experimental design matrix and results were carried out as per Table 3. Design-Expert 8.0.6 software was used for data design of CCD-RSM and optimal formulation was predicted. Concentrations of ethanol (A), CHO (B), and SPC (C) were selected as independent variables while EE, ZP, VS, and PDI were selected as the dependent variables. The desirability function for the response to be minimized was defined as [27]:

(2) $$di_{\min}=\frac{Y_{\max}-Y_{i}}{Y_{\max}-Y_{\min}}$$

For a response to be maximized, the desirability function was defined as:

(3) $$di_{\min}=\frac{Y_{i}–Y_{\min}}{Y_{\max}–Y_{\min}}$$

where Y_min_, Y_max_, and Y_i_ represented the lowest possible value, highest possible value, and the experimental value, respectively. After the individual desirability of each response was determined, an overall desirability could be calculated using the formula:

OD = (d_1_d_2_……di)^1/k^, (4)

where k was the number of responses.

### 4.3. Characterization of PAE Ethosomes

#### 4.3.1. Determination of Drug EE

EE was determined by the ultrafiltration centrifugation method, using regenerated cellulose filters with molecular exclusion pore size of 10 kDa (Millipore). The quantities of paeonol in the non-encapsulated form in the filtrates and in vesicular suspension were analyzed by high-performance liquid chromatography (HPLC) instrument (Agilent Technologies 1260 series, Waldbronn, Germany). Encapsulation efficiency (EE) was calculated by: [(Dp − Ds)/Dp] × 100%, where Dp is the total amount of paeonol in the vesicular suspension and Ds is the free drug content in the supernatant.

#### 4.3.2. Determination of VS, PDI and ZP

The average vesicle size (VS), polydispersity index (PDI), and zeta potential (ZP) of the PAE ethosomes were measured at 25 °C using a Zetasizer Nano ZS90 (Malvern Instruments, Malvern, UK). Mean value of three repeated measurements of each sample was reported as the final result.

#### 4.3.3. Morphological Study by Transmission Electron Microscopy (TEM)

Morphology of the vesicles was characterized by transmission electron microscopy (JEM-1230, JEOL, Akishima, Japan). For TEM, samples were negatively stained with 1% phosphotungstic acid on a copper grid and allowed to dry at room temperature.

#### 4.3.4. Differential Scanning Calorimetry (DSC)

DSC was used to investigate the physical state of drug and lipid when entrapped within the ethosomal vesicles. In order to ensure a smooth process of freeze-drying, mannitol (5% *w*/*v*) was added as a freeze-drying protective agent. PAE ethosomes were prefrozen for 24 h in ultralow-temperature refrigerator at −80 °C, followed by freeze-drying (−50 °C, −0.01 MPa) for 72 h, eventually resulting in the freeze-dried PAE ethosome powder. The thermal properties of pure paeonol, CHO, SPC, mannitol, physical mixture, and freeze-dried PAE ethosome powder were investigated using DSC (Mettler Toledo TGA/DSC 3+, Schwerzenbach, Switzerland). A temperature range between 10 °C and 400 °C was selected for thermal scanning of the samples at a heating rate of 5 °C/min, under a constant flow of nitrogen gas.

#### 4.3.5. X-ray Diffraction (XRD)

XRD was used to investigate the amorphous phase of the drug within the formulations. Different samples (pure paeonol, CHO, SPC, mannitol, physical mixture, and freeze-dried PAE ethosome powder) were investigated by the Bruker-AXS D8 Advance diffractometer (Karlsruhe, Germany) with a Cu K radiation source fixed at 40 kV and 100 mA. XRD was performed at the scanning rate of 1°/s over a scanning range 2θ of 10–70 degrees.

#### 4.3.6. Fourier-Transform Infrared Spectroscopy (FT-IR)

Drug–excipient compatibility studies were carried out using Fourier-transform infrared spectroscopy (FT-IR) with the Tensor 27 (Bruker, Karlsruhe, Germany) on KBr disks. The study was carried out on individual components: pure paeonol, CHO, SPC, mannitol, physical mixture, and freeze-dried PAE ethosome powder. The resulting FT-IR spectra were analyzed based on the intensity and shift of vibration bands.

### 4.4. HPLC Assays for Paeonol

In this study, paeonol was quantified by HPLC using an Agilent 1260 system equipped with a UV detector (Agilent DEA601776). The analysis was performed using a Thermo Scientific C18 column (250 × 4.0 mm i.d., 5-μm particle size). The prepared sample solution was filtered through a 0.45-μm membrane prior to injection into the HPLC column. The column was maintained at 30 °C; the mobile phase was composed of methanol:water (60:40) with a flow rate of 0.8 mL/min.

### 4.5. Ex-Vivo Animal Studies

#### 4.5.1. Preparation of the Skin

Prior to the study, rat fur (from male rats, approximately 250 g each) was carefully trimmed and shaved by an electric shaving machine, and the dorsal skin was separated from the underlying connective tissue with a scalpel.

#### 4.5.2. In-Vitro Transdermal Absorption Study

The in-vitro skin permeation of paeonol from PAE ethosomes was studied using Franz diffusion cells with an effective permeation area of 0.785 cm^2^. The skin was mounted between the donor and receptor compartments, while keeping the stratum corneum toward the donor compartment. Ethanol (25% *v*/*v*) was taken as receiver medium and kept at 37 ± 0.5 °C, using a water bath, under constant magnetic stirring for 24 h. Ethosomal formulation (1 mL) was applied to the epidermal surface of skin and covered with parafilm to avoid evaporation. Aliquots of 0.5 mL receptor medium at the time points 0.5, 1, 2, 4, 6, 8, 12, and 24 h were removed, and replaced by an equal volume of fresh medium. The paeonol content in 0.5-mL receptor medium was filtered and analyzed by HPLC; the cumulative penetration amount of paeonol per cm^2^ of diffusion area through the skin (Qn) was calculated according to equation:

(5) $$Q_{n}=\frac{VC_{n}+\sum_{i-1}^{n-1}V_{i}C_{i}}{S},$$

where C_n_ stands for the paeonol concentration in receiver medium at time n, C_i_ is the drug concentration of the sample, S is the effective diffusion area (0.785 cm^2^), and V and Vi represent the volumes of receiver solution (5 mL) and sample (0.5 mL), respectively.

#### 4.5.3. In-Vitro Skin Retention Study

To estimate drug retention in the skin, at the end of the skin permeation experiment, excess paeonol was wiped gently from the surface of the skin with filter paper, and the skin surface washed with methanol and normal saline and dried with filter paper. The skin was cut into small pieces, 1.0 mL of methanol was added to them, they were homogenized for 10 min, and centrifuged at 7000 rpm for 10 min. Subsequently, the supernatant was collected and filtered for HPLC.

### 4.6. In-Vivo Skin Irritation Study

Histopathology of skin was conducted to visualize the topical effect of PAE ethosomes on skin. Group 1 was exposed to PAE ethosomes, whereas group 2 was taken as a control. The formulation was applied to the rat skins and covered with a parafilm patch held by a bandage for 24 h. The rats were subsequently decapitated and the treated skins removed. The skin samples were collected in 10% formalin for tissue fixation for 24 h. Thereafter, the skins were washed in warm water and vertically cut into 3–4 µm thick sections. The paraffin tissue sections were subjected to hematoxylin–eosin staining, followed by microscopic examination for any histological change. The tests were performed on three rats for each group.

### 4.7. Physical Stability Assay

Physical stability of the vesicles was determined by storing the vesicles at 4 ± 1 °C and 25 ± 1 °C for 30 days. During the storage period, EE, ZP, VS, and PDI were measured at 0, 10, 20, and 30 days.

### 4.8. Statistical Analysis

Statistical significance of differences was determined using one-way analysis of variance (ANOVA); a value of *p* < 0.05 was considered to be significant.

## 5. Conclusions

In the present study, we established a novel transdermal vesicular delivery system for paeonol, PAE ethosomes. The PAE ethosomes were prepared by the injection method, and optimized by the single-factor test and central composite design-response surface methodology. The optimized PAE ethosomes were characterized in terms of EE, VS, ZP, PDI and morphology under TEM. Additionally, DSC, XRD and FT-IR studies revealed the amorphous state of paeonol in ethosomes, indicating successful incorporation of paeonol into ethosomes. Results of in-vitro transdermal absorption and skin retention studies indicated that the cumulative penetration (Qn) and deposition (Qs) of paeonol from the ethosomes were significantly higher than from 25% hydroethanolic solution, hence indicating not only enhanced transdermal absorption by ethosomes, but also increased storage in the skin. Histopathologic studies proved that the prepared PAE ethosomes were safe and available for application to skin. The ethosomes composed of ethanol, SPC and CHO had optimum properties, good skin permeability and acceptable stability, which makes ethosomes a prospective route for paeonol.

## Figures and Tables

**Table 1:** Arrangement and determination results of single-factor test (mean ± SD, *n* = 3).

Factor	Level	EE (%)	ZP (−mV)	VS (nm)	PDI	OD
**Ethanol** **(% *w*/*v*)**	15%	75.13 ± 1.08	11.10 ± 1.05	123.7 ± 9.5	0.213 ± 0.003	0.72 ± 0.02
	20%	76.33 ± 1.11	12.31 ± 1.09	145.7 ± 4.7	0.262 ± 0.012	0.77 ± 0.04
	25%	72.56 ± 2.02	14.00 ± 1.11	175.6 ± 7.5	0.283 ± 0.006	0.79 ± 0.02
	30%	65.85 ± 1.06	11.71 ± 1.15	201.1 ± 9.3	0.308 ± 0.021	0.53 ± 0.10
	35%	53.63 ± 2.03	12.52 ± 1.54	215.8 ± 8.8	0.295 ± 0.032	0.54 ± 0.09
	40%	34.26 ± 1.13	10.00 ± 1.77	399.7 ± 10.4	0.360 ± 0.034	0.00 ± 0.00
**CHO** **(% *w*/*v*)**	0%	76.1 ± 1.05	6.45 ± 1.11	117.2 ± 4.8	0.256 ± 0.014	0.00 ± 0.00
	1%	77.31 ± 2.10	13.00 ± 1.83	138.5 ± 5.2	0.271 ± 0.032	0.65 ± 0.04
	2%	77.58 ± 2.08	13.30 ± 0.36	140.3 ± 8.4	0.290 ± 0.011	0.51 ± 0.13
	3%	78.21 ± 3.05	9.17 ± 1.32	148.0 ± 9.6	0.286 ± 0.025	0.47 ± 0.12
	4%	77.30 ± 0.56	8.83 ± 1.20	184.1 ± 9.2	0.289 ± 0.055	0.21 ± 0.23
	5%	79.18 ± 1.35	8.28 ± 0.64	188.6 ± 9.9	0.298 ± 0.068	0.00 ± 0.00
**SPC** **(% *w*/*v*)**	1%	57.42 ± 0.24	19.70 ± 1.12	100.4 ± 3.6	0.241 ± 0.021	0.00 ± 0.00
	2%	70.56 ± 2.25	18.60 ± 1.24	114.1 ± 5.5	0.285 ± 0.008	0.70 ± 0.08
	3%	78.80 ± 1.29	15.70 ± 0.67	127.5 ± 7.2	0.302 ± 0.022	0.64 ± 0.18
	4%	83.27 ± 0.78	14.10 ± 0.35	139.5 ± 8.6	0.338 ± 0.043	0.51 ± 0.16
	5%	83.65 ± 2.22	12.90 ± 1.44	176.6 ± 9.3	0.373 ± 0.056	0.29 ± 0.17
	6%	87.38 ± 1.32	11.87 ± 0.23	201.0 ± 11.5	0.415 ± 0.059	0.00 ± 0.00

Note: EE, ZP, VS and PDI means encapsulation efficiency, vesicle size, zeta potential and polydispersity index, respectively.

**Table 2:** Independent variables and the corresponding levels in central composite design.

Variables	Levels		
	−1	0	1
A/%	15	20	25
B/%	0.5	1.0	1.5
C/%	1.5	2.0	2.5

Note: A (the concentrations of ethanol), B (the concentrations of CHO), C (the concentrations of SPC).

**Table 3:** Experimental design matrix and results.

Run	A (%)	B (%)	C (%)	EE (%)	ZP (−mV)	VS (nm)	PDI	dEE	dZP	dVS	dPDI	OD
1	25	1.5	2	84.65	15.4	113.4	0.138	0.97	0.61	0.95	0.78	0.81
2	20	1.5	1.5	67.54	18.1	145.5	0.142	0	1	0	0.73	0
3	20	0.5	1.5	75.31	11.5	111.6	0.208	0.44	0.06	1	0	0
4	20	1	2	77.98	15.8	119.9	0.127	0.59	0.67	0.76	0.9	0.72
5	20	1	2	80.25	13.9	113.5	0.124	0.72	0.4	0.94	0.93	0.71
6	25	0.5	2	80.69	13.2	137.5	0.134	0.75	0.3	0.24	0.82	0.46
7	15	0.5	2	78.36	12.9	143.5	0.151	0.62	0.26	0.06	0.63	0.28
8	25	1	2.5	85.12	17.2	120.8	0.141	1	0.87	0.73	0.74	0.83
9	20	1	2	76.98	15.5	119.4	0.129	0.54	0.63	0.77	0.88	0.69
10	20	0.5	2.5	79.48	12.2	132.9	0.156	0.68	0.16	0.37	0.58	0.39
11	25	1	1.5	71.21	17.5	140.3	0.118	0.21	0.91	0.15	1	0.41
12	15	1.5	2	80.55	15.8	142.6	0.165	0.74	0.67	0.09	0.48	0.38
13	15	1	1.5	78.97	11.1	140.6	0.141	0.65	0	0.14	0.74	0
14	20	1.5	2.5	82.46	16.1	126.3	0.146	0.85	0.71	0.57	0.69	0.7
15	20	1	2	81.89	15.7	123.5	0.125	0.82	0.66	0.65	0.92	0.75
16	20	1	2	78.31	15.8	120.9	0.125	0.61	0.67	0.73	0.92	0.72
17	15	1	2.5	77.48	16.4	124.1	0.133	0.57	0.76	0.63	0.83	0.69

Note: dEE, dZP, dVS and dPDI represents the desirability functions of EE, ZP, VS and PDI, respectively.

**Table 4:** Characterization of PAE ethosomes (mean ± SD, *n* = 3).

Code	EE (%)	ZP (−mV)	VS (nm)	PDI	OD
Actual values	84.33 ± 1.34	16.8 ± 0.36	120.2 ± 1.3	0.131 ± 0.006	0.84 ± 0.02
